# Nanosecond Time Synchronization over a 2.4 GHz Long-Range Wireless Link

**DOI:** 10.3390/s25071961

**Published:** 2025-03-21

**Authors:** Pascal Müller, Dominic Berger, Luciano Sarperi

**Affiliations:** Institute of Signal Processing and Wireless Communications, ZHAW School of Engineering, 8401 Winterthur, Switzerland; pascal.mueller@zhaw.ch (P.M.); dominic.berger@zhaw.ch (D.B.)

**Keywords:** time synchronization, wireless synchronization, long-range (LoRa) modulation, nanosecond accuracy

## Abstract

Time synchronization between geographically separated equipment, such as, for example, that required in sensor networks for radio localization, is often based on global navigation satellite systems (GNSSs). However, in environments that are GNSS-denied due to signal blockage or interference, alternative timing synchronization methods are necessary. In this work, an experimental wireless time synchronization system based on long-range (LoRa) modulation has been developed and tested in the field. LoRa modulation operating in the license-free 2.4 GHz industrial, scientific and medical (ISM) band was chosen due to the potentially large coverage area of several kilometers and the availability of a ranging engine in the SX1280 transceiver by Semtech, which facilitates the implementation of time synchronization. The prototype system was tested over 170 m, where it achieved a time deviation (TDEV) of 30 ps for an average time of 1 s and a maximum TDEV of 3 ns over one day of measurement, improving over existing work on time synchronization with LoRa modulation by around three orders of magnitude. The field tests showed that ns accuracy can be achieved using LoRa modulation, making it suitable for the synchronization of remote sites, for example, for radio localization.

## 1. Introduction

Time synchronization plays an important role in many systems and applications, such as in cellular networks for frequency and time synchronization between base stations, in distributed sensor networks for the timestamping of data or for radio localization using the time difference of arrival method [1].

A typical way of achieving time synchronization between two points is using global navigation satellite systems (GNSSs) [2]. The accuracy of a 1 Hz pulse per second (PPS) can reach 200 ps with additional signal conditioning [3]. However, when GNSS signals are not available due to blocked lines of sight from the receiver to the satellites or when external interference is present, other time synchronization methods are required.

When cables can be used, high-accuracy clock synchronization may be implemented with White Rabbit (WR) time synchronization technology, which is based on optical ethernet networks. WR is an extension of the Precision Time Protocol (PTP) and has been incorporated into the IEEE 1588 standard. It uses a PHY-layer frequency synchronization of the involved nodes and phase measurements, thereby achieving accuracies in the sub-ns level [4].

However, when synchronization between ad hoc nodes is required and GNSSs cannot be used, alternative, wireless methods for time synchronization are desirable. Time synchronization over optical free space links can achieve remarkably high accuracies, with reported values in the sub-ps level for a 113 km link [5]. However, optical free-space links require a line-of-sight path and are susceptible to adverse weather conditions such as precipitation, fog or turbulent air, with an additional link loss that may exceed 100 dB/km in case of fog [6], which can render the operation impossible.

Compared to optical free-space links, wireless time synchronization systems using radio frequency (RF) signals are less influenced by atmospheric conditions and can, in principle, also be employed without a direct line-of-sight link between the two terminals. There is a large amount of previous work on wireless time synchronization for sensor networks, with typical accuracies in the µs level, for example [7,8]. Other work on wireless time synchronization focuses on short-range wireless time synchronization, for example, using ultra-wideband (UWB) technology, with reported accuracies in the ns range [9]. In [10], a wireless system for positioning and time synchronization operating in the 2.4 GHz industrial, scientific and medical (ISM) band is evaluated, with a reported maximum time interval error (MTIE) of 3 ns averaged over multiple distances of up to 105 km.

In the area of wireless time synchronization with long-range (LoRa) modulation, existing work focuses either on the actual time synchronization of devices or on methods for timestamping events or data packets. In the following, previous work on the time synchronization of LoRa devices is reviewed, which is related to our work. In [11,12], an energy-efficient synchronization method with low-layer timestamping based on interrupt signals is proposed, which achieves an accuracy of 15 μs. However, since it uses a one-way exchange, the propagation delay cannot be compensated. In [13], a time synchronization method for LoRaWAN networks based on low-layer interrupt signals is proposed. It uses either one two-way exchange to adjust local clocks and perform a first-order compensation of clock drift rate or two two-way exchanges to additionally compensate the propagation delay. With one two-way exchange, an accuracy of 3 μs is achieved, while, with two two-way exchanges over distances of up to 4 km, an accuracy of 10 μs is achieved. The method in [14] presents a time synchronization method for LoRa devices with the aim of optimizing the channel access by introducing scheduling to increase the system capacity. The proposed time synchronization protocol, which does not use low-layer timestamps, has a time resolution of 10 ms. In [15], a synchronization method for LoRa devices using the ranging function of the SX1280 transceiver from Semtech is proposed, which is used to compensate the propagation delay. Few details about the measurement setup are provided, but an accuracy of about 1 μs for distances of 100 m is reported.

Recent work on the timestamping of events or data packets in LoRa networks includes [16,17]. In [16], a synchronization-free timestamping of uplink data at the LoRa gateway with ms accuracy is proposed, while, in [17], a posteriori time synchronization for LoRa devices is proposed, which can be used for the timestamping of events with ms accuracy. Since, in both works, no actual synchronization between devices is performed, these approaches are not directly applicable to time synchronization.

The aim of this work is the wireless time synchronization of a remote device over potential distances of several kilometers with ns accuracy using LoRa modulation. In [18], we investigated the use of LoRa modulation for outdoor ranging and positioning, which we extend here to time synchronization. LoRa modulation is popular for low-data-rate IoT applications due to its large coverage of several kilometers and low energy consumption [19]. Compared to previous work on LoRa time synchronization in [11,12,13,14], we employ the ranging engine of the SX1280 transceiver from Semtech, which is intended for distance measurements. The ranging engine uses a two-way exchange with dedicated ranging packets containing 15 ranging symbols. Although no implementation details of the ranging engine are provided in the data sheet [20], a best-case standard deviation for distance measurement with the Development Kit of 0.4 m is reported in the application note [21], corresponding to 1.3 ns. This makes the SX1280 a suitable platform for implementing an experimental wireless time synchronization system with the aim of ns accuracy. Although [15] also uses the ranging engine of the SX1280, the system is implemented with a lower clock resolution, resulting in significantly lower accuracy. Compared to the method in [10], which does not use LoRa modulation but obtains similar performance to our system, we measure and compensate the time of flight between the two terminals, making it more suitable for ad hoc applications. To the best of our knowledge, this work is the first application of LoRa modulation for time synchronization that achieves ns accuracy.

## 2. Time Synchronization with LoRa Modulation

### 2.1. LoRa Modulation

**Figure 1:**
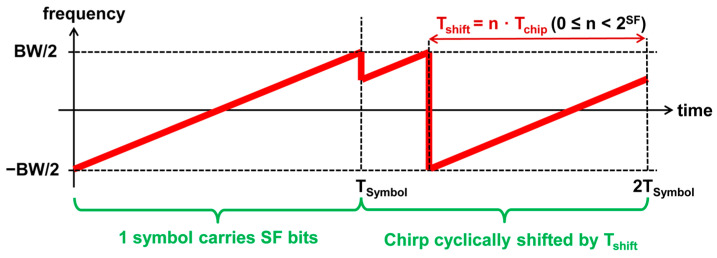
LoRa modulation.

Long-range (LoRa) modulation is a chirp spread spectrum (CSS) modulation that was developed by the Semtech Corporation [22] and has become popular for IoT applications. Each symbol consists of a cyclically shifted frequency ramp with a bandwidth BW, as shown in Figure 1.

The information to be transmitted is modulated on the symbol by $2^{SF}$ possible cyclic time shifts $T_{shift}$ of the frequency ramp, whereby a symbol carries SF bits. The value SF is also termed spreading factor but does not play the same role as in code-division multiple-access (CDMA) systems, since the bandwidth can be configured independently of the spreading factor. The cyclic shift $T_{shift}$ has a resolution of $T_{chip}=1/BW$.

LoRa modulation is typically used in the license-free 863–870 MHz band in Europe and 902–928 MHz in North America. However, in Europe, the duty cycle is limited to 1 % and the effective radiated power (ERP) is limited to 14 dBm in most of the 863–870 MHz frequency range [23]. Since this duty cycle limitation would not allow for a regular packet exchange as required for time synchronization, in this work, the 2.4 GHz ISM frequency band was used for implementing the time synchronization system, where no duty cycle limitation is in place.

The SX1280 transceiver of Semtech was chosen, since it operates in the 2.4 GHz ISM frequency band and incorporates a ranging engine, which is primarily intended to measure the time of flight between two SX1280 transceivers [20]. The SX1280 supports bandwidths BW=406, 812 and 1625 kHz and spreading factors SF=5, 6, …, 10 for ranging. With the maximum bandwidth of 1625 kHz, a distance resolution of

(1) $$d_{R}=\frac{c}{BW}=\frac{3\cdot 10^{8}}{1625\cdot 10^{3}}=185\;m$$

can be achieved; see Section 2.1.3 in [24]. The distance resolution translates into a time-of-flight resolution of $t_{TOF}=d_{R}/c=185/3\cdot 10^{8}=617\;ns$, which seems to be too large for a time synchronization system with a targeted accuracy in the ns range. However, since the time synchronization system must not resolve different multipaths but rather compensate the time of flight as seen by the system between the transmitter and receiver, it is possible to achieve a higher time synchronization accuracy than the limited temporal resolution provided by the relatively small signal bandwidth. This is shown by the time deviation of 3 ns obtained by the proposed system in Section 3.2, which is significantly lower than the time-of-flight resolution $t_{TOF}=617\;ns$. However, the accuracy will depend on the signal-to-noise ratio (SNR) [25].

Regarding the achievable range, the best RX sensitivity of −122 dBm is obtained with BW=406 kHz and SF=10, resulting in a maximum coupling loss of 134.5 dB for the maximum TX power of 12.5 dBm supported by the SX1280 ranging engine [20]. This corresponds to a range of several kilometers in a rural non-line-of-sight environment with omni-directional antennas.

### 2.2. Time Synchronization Protocol and Method

**Figure 2:**
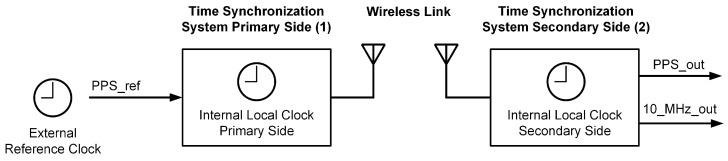
Experimental wireless time synchronization system.

The time synchronization is based on the SX1280 PHY-layer ranging engine, which uses a two-way exchange for half-duplex systems according to Section 6.1 of [24] to determine the time of flight (TOF) between two SX1280 devices. With a two-way exchange, it is also possible to measure the clock offset between two terminals with unsynchronized clocks, which will be used here to align the clock of the secondary side to the reference clock on the primary side [26]. Figure 2 shows an overview of the developed time synchronization system. The external reference clock in the form of a 1 Hz PPS signal is input to the primary side, where it is used to synchronize the internal local clock to provide local timestamps. Using the subsequently described time synchronization protocol, the internal local clock on the secondary side is synchronized in frequency and phase to the primary side. The synchronized time is then provided as a 1 Hz PPS signal, with an additional 10 MHz reference frequency signal.

**Figure 3:**
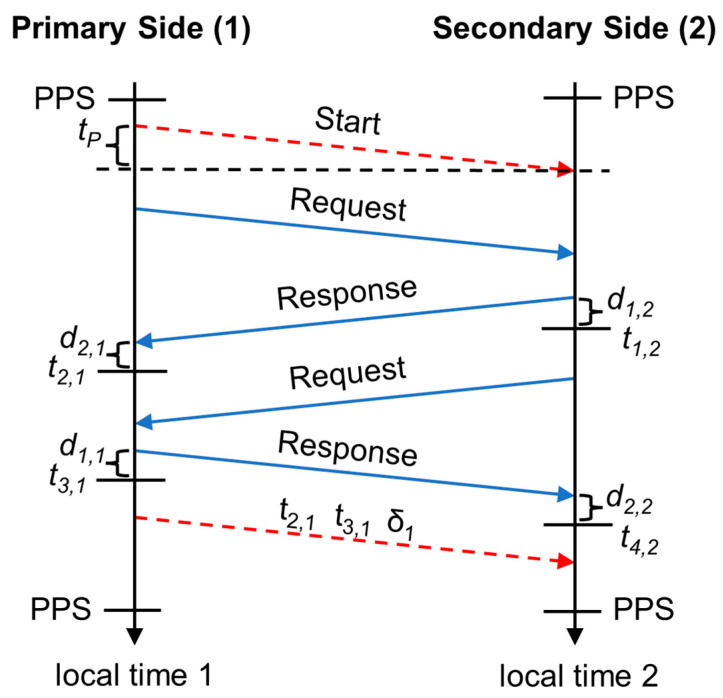
Ranging exchanges used for time synchronization.

In Figure 3, two sequential two-way exchanges between the primary and secondary side are shown, each consisting of a request, followed by a response after a fixed delay. The ranging engine of the SX1280 transceiver is primarily designed to measure the range between two devices based on the round-trip time of flight, which it determines using a single two-way exchange. To perform time synchronization with a single two-way exchange, both the transmit and receive interrupts (to determine a transmission and reception timestamp) of each of the two messages are required. However, the SX1280 ranging engine only provides transmit and receive interrupts for the response.

Therefore, we use two sequential two-way ranging exchanges of the SX1280 to mimic a single two-way ranging exchange to provide the four required timestamps. By defining the timestamp $t_{a,b}$ in Figure 3 as the timestamp number a on side b, the transmit and receive interrupts are then used to obtain the corresponding four timestamps $t_{1,2}$, $t_{2,1}$, $t_{3,1}$ and $t_{4,2}.$ To mark the beginning of the ranging exchanges, we use a start packet. Additionally, a packet with timestamps $t_{2,1}$, $t_{3,1}$ and the normalized local clock frequency $\delta_{1}$ is sent from the primary to the secondary side at the end of the ranging exchanges, as described subsequently.

In the first ranging exchange, started from the primary side according to Figure 3, the timestamp number 1 at the secondary side (2) $t_{1,2}$ is measured, as well as the timestamp number 2 at the primary side (1) $t_{2,1}$. These two timestamps correspond to the transmission time of the response plus an internal delay $d_{1,2}$ and to the reception time of the response plus an internal delay $d_{2,1}$ on side 2 and side 1, respectively. In the second ranging exchange, started from the secondary side, the timestamps number 3 and 4 are measured, at the primary side (1) as $t_{3,1}$ and at the secondary side (2) as $t_{4,2}$. These timestamps also include their corresponding internal delays.

The timestamps are measured using local unsynchronized clocks (a free running timer on the primary and secondary side), as the controller only moves the position of the one-second period start to synchronize the local clock on both sides. The one-second period start corresponds to the positions of the PPS marker shown in Figure 3. This PPS marker corresponds on the secondary side to the synchronized time information. It should be noted that Figure 3 shows the synchronized state, where the PPS marker positions of the primary and secondary side have been aligned. Therefore, the local timestamps from the free running timers must be corrected to reflect the correction of the control algorithm on the local clock. For the local time on the primary side (1) as an example, this corrected time is

(2) $$t_{2,1corr}=\frac{t_{2,1}}{\delta_{1}}$$

in which the locally measured unsynchronized timestamp 2 on the primary side 1, expressed as $t_{2,1}$, is translated into timestamp 2 with the correction of the control algorithm $t_{2,1corr}$. The correction value is the normalized frequency of the timer on the primary side,

(3) $$\delta_{1}=1+\frac{\Delta f_{1}}{f_{nominal}},$$

where $\Delta f_{1}$ is the frequency error and $f_{nominal}$ is the nominal frequency of the local timer. The local time on the secondary side can be corrected in an analogous way. Equation (2) may also be rewritten to calculate local unsynchronized times from corrected times.

Time synchronization can be achieved by minimizing the time error ${∆t}_{2}$ between the timestamp $t_{1,2}$ in the local unsynchronized time of side 2 and the nominal timestamp $t_{1,2,nominal}$, which is also in the local unsynchronized time 2 but calculated from the reference time as shown subsequently. The same time offset can also be expressed using the timestamp $t_{4,2}$, resulting in

(4) $${∆t}_{2}=t_{1,2}-t_{1,2,nominal}=t_{4,2}-t_{4,2,nominal}.$$

The measured value of ${∆t}_{2}$ is used as the control deviation by the controller of the local timer to adjust the position of the 1 s PPS marker. Using the correction in Equation (2) and by introducing the signal propagation time $t_{p}$ between the two devices, the two nominal timestamps according to Figure 3 become

(5) $$t_{1,2,nominal}=\left(\frac{t_{2,1}}{\delta_{1}}-\frac{d_{2,1}}{\delta_{1}}-t_{p}+\frac{d_{1,2}}{\delta_{2}}\right)\delta_{2}$$

(6) $$t_{4,2,nominal}=\left(\frac{t_{3,1}}{\delta_{1}}-\frac{d_{1,1}}{\delta_{1}}+t_{p}+\frac{d_{2,2}}{\delta_{2}}\right)\delta_{2},$$

where $t_{p}$ corresponds to the propagation delay plus any hardware delays (due to transmission lines, filters and the low-noise amplifier on the printed circuit board and RF cables). Since the channel is assumed to remain static during the two sequential two-way ranging exchanges, the delay $t_{p}$ is constant. Additionally, $t_{p}$ is assumed to be identical in both directions.

Combining Equations (4)–(6) produces

$${∆t}_{2}=\frac{1}{2}\left(t_{1,2}-t_{1,2,nominal}\right)+\frac{1}{2}\left(t_{4,2}-t_{4,2,nominal}\right)=$$

(7) $$\frac{1}{2}\left[t_{1,2}-\left(\frac{t_{2,1}}{\delta_{1}}-\frac{d_{2,1}}{\delta_{1}}+\frac{d_{1,2}}{\delta_{2}}\right)\delta_{2}+t_{4,2}-\left(\;\frac{t_{3,1}}{\delta_{1}}-\frac{d_{1,1}}{\delta_{1}}+\frac{d_{2,2}}{\delta_{2}}\right)\delta_{2}\right].$$

Although the datasheet of the SX1280 [20] does not specify the following values and they are not accessible to the user, based on our observations, we assume for the delays that $d_{1,1}=d_{1,2}$ and $d_{2,1}=d_{2,2}$ hold and that the values are <20 μs. Furthermore, with the chosen local oscillator, the normalized frequency errors are guaranteed to fulfill $\epsilon_{1}={\Delta f}_{1}/f_{nominal}<\pm 1\;\mathrm{ppm}$ and $\epsilon_{2}=\Delta f_{2}/f_{nominal}<\pm 1\;\mathrm{ppm}$. With these assumptions, we obtain

(8) $$\left|\frac{d_{2,1}}{\delta_{1}}-\frac{d_{2,2}}{\delta_{2}}\;\right|<40\;\mathrm{ps},$$

which is significantly smaller than the desired accuracy. An analogous statement can also be made for $d_{1,1}$ and $d_{1,2}$. With the above assumptions, the terms containing the delays $d_{2,1}$, $d_{2,2}$, $d_{1,1}$ and $d_{1,2}$ in Equation (7) are negligible. Consequently, the time offset in Equation (7) simplifies to

(9) $${∆t}_{2}\approx \frac{1}{2}\left[t_{1,2}-\left(\frac{t_{2,1}}{\delta_{1}}\right)\delta_{2}+t_{4,2}-\left(\;\frac{t_{3,1}}{\delta_{1}}\right)\delta_{2}\right],$$

which expresses ${∆t}_{2}$ in terms of uncorrected local times on the secondary side 2. Equation (9) can be converted to the corrected local times on the secondary side 2 based on Equation (2) as

(10) $${{∆t}_{2}}_{corr}=\frac{{∆t}_{2}}{\delta_{2}}\approx \frac{1}{2}\left(\frac{t_{1,2}}{\delta_{2}}-\frac{t_{2,1}}{\delta_{1}}+\frac{t_{4,2}}{\delta_{2}}-\frac{t_{3,1}}{\delta_{1}}\right).$$

Since the goal of the time synchronization is to minimize the time offset between the secondary and primary side, either Equation (9) or (10) may be used as a control input for adjusting the position of the 1 s PPS marker.

Equation (10) corresponds to a standard two-way ranging procedure [24], which is started from the secondary side, except for the addition of the time corrections. The correction values $\delta_{1}$ and $\delta_{2}$ are determined from the integral components of the employed proportional–integral (PI) controllers on the primary side 1 and secondary side 2, respectively, as described in Section 2.3.2. The value $\delta_{1}$ is transmitted at the end of the ranging exchanges together with the stamps $t_{2,1}$ and $t_{3,1}$ from the primary to the secondary side, as shown in Figure 3. The initial values of $\delta_{1}$ and $\delta_{2}$ are set to zero.

### 2.3. Time Synchronization System Design

The following subsections describe the design and implementation of the wireless time synchronization system. This section is structured as follows. The first subsection provides an overview of a time synchronization device configured for primary-side and for secondary-side operation. The second subsection explains the measurement of the timestamp values $t_{1,2}$, $t_{2,1}$, $t_{3,1}$ and $t_{4,2}$, as well as the 1 Hz PPS signal generation. Next, the phase offset and cable delay compensation are described. The last subsection gives an overview of the implemented LoRa communication protocol and timing.

#### 2.3.1. Overview

**Figure 4:**
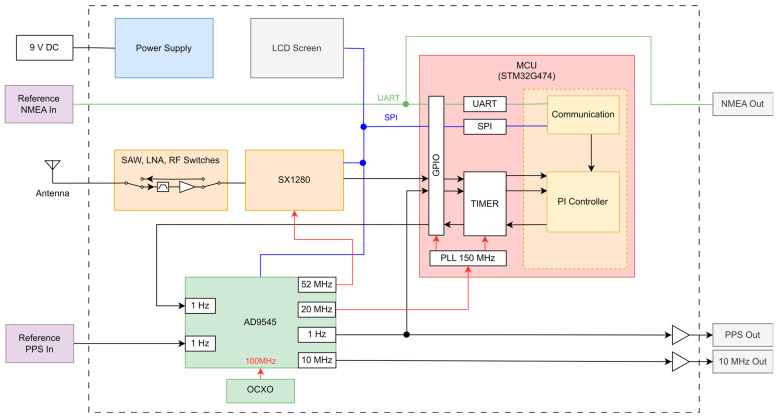
Wireless time synchronization system block diagram (primary-side and secondary-side operation shown).

Figure 4 shows the block diagram of our wireless time synchronization system based on the LoRa transceiver SX1280. Starting at the antenna, an SAW filter and low-noise amplifier are used in the receive direction to improve the immunity against out-of-band interference and to reduce the total noise figure. The necessary RF switches to change between the receive and transmit direction are controlled by the MCU.

The STM32F474 microcontroller unit (MCU) contains the application that covers the tasks for communication protocol handling, including timestamp measurement, the PPS controller and the low-level hardware drivers, as well as the visualization of the network and time synchronization status on a display. National Marine Electronics Association (NMEA) compliant time and date information can be exchanged with the help of the RS232 and USB interfaces.

The internal and external clock signals are generated within an Analog Devices AD9545 “1 PPS synchronizer and jitter cleaner” using an Abracon AOCJY3A 100 MHz oven-controlled crystal oscillator (OCXO) with a rated frequency stability of 10 ppb. The 52 MHz clock for the LoRa transceiver and the 20 MHz clock for the MCU are generated from a primary analogue phase-locked voltage-controlled oscillator (VCO) that is linked to the OCXO, while the 1 Hz PPS and 10 MHz output signals are derived from a secondary phase-locked loop (PLL) with a VCO running at 2.46 GHz. This secondary PLL digitally compares the divided 1 Hz PPS output with a 1 Hz reference input and disciplines the underlying analogue PLL, which is also linked to the OCXO. This secondary PLL contains a 10 mHz loop filter used to clean up the signal by reducing the jitter and quantization noise of the output 1 Hz PPS signal. The OCXO and AD9545 are the main energy consumers, having a total steady state power consumption of 3 W.

The secondary PLL configuration depends on the device operating mode. For the primary side, the frequency and phase synchronization are based on the external 1 Hz PPS signal of the reference clock. The cleaned-up internal 1 Hz PPS signal output of the DPLL is then synchronous to the external reference clock and is measured by an internal 32-bit hardware timer of the MCU. This measurement is used to synchronize the internal local clock of the MCU, which serves as the time base for the application. Due to the limited counting frequency of 150 MHz for the timer, a time quantization error of up to ±3.33 ns arises. This is also valid for the timestamp measurement of the interrupt signals $t_{1,2}$, $t_{2,1}$, $t_{3,1}$ and $t_{4,2}$ generated by the LoRa transceiver.

The secondary side uses the same timer to generate the 1 Hz PPS signal of the local clock based on the timestamps of the ranging exchanges using a PI controller. The secondary PLL uses this 1 Hz PPS signal as reference and reduces its quantization error to the sub-ns range in the 1 Hz PPS output signal.

#### 2.3.2. Timer-Based Timestamp Measurement and 1 Hz PPS Generation

An internal 32-bit hardware timer of the MCU running at 150 MHz is used to measure the timestamps of the interrupt signals and to generate the output 1 Hz PPS signal of the MCU on the secondary side. The timer clock signal is derived from the OCXO without frequency offset correction. It is configured to count from 0 to P−1 and restarts again from 0, where P is the overflow or 1 Hz period value. P is initially set to $P_{N}=150\cdot 10^{6}$, which corresponds to the nominal duration of one period of the 1 Hz PPS signal.

The frequency and phase offset measurement between the 1 Hz PPS signal of the external reference clock cleaned up by the DPLL and the internal local clock on the primary side is performed with one input capture/compare channel. The first 1 Hz PPS timestamp measurement is used to correct the coarse phase offset by adapting the overflow value P for one period and then switches back to ${P=P}_{N}$. The following measurements are then expected to be within some microseconds before or after an overflow (corresponding to the positions of the PPS marker shown in Figure 3) and are fed as time error values $\Delta t_{ref}$ to the PI controller. Small positive values are measured when an overflow event occurs before receiving the reference 1 Hz PPS signal, which indicates a too-high local oscillator frequency and vice versa.

The PI controller is responsible for adapting the overflow value P to align the phase of the PPS Out signal to the phase of the reference signal and increases P in case of a too-high local oscillator frequency to compensate the deviation. This is implemented according to

(11) $$P=P_{N}+K_{P}\Delta t_{2}+K_{I}\int_{0}^{t}\Delta t_{ref},$$

where $K_{P}=0.05$ and $K_{I}=0.005$ are heuristically determined constants.

The normalized local clock frequency error can be determined by

(12) $$\epsilon =\frac{P}{P_{N}}-1=\frac{\Delta f}{f_{nominal}}$$

and the correction value required for the time synchronization method in Section 2.2 corresponds to

(13) $$\delta =\epsilon +1=\frac{P}{P_{N}}$$

where δ can stand for $\delta_{1}$ or $\delta_{2}$ depending on the device operation mode.

The secondary side uses the calculated time error $\Delta t_{ref}={∆t}_{2}$ from (9) as input to the PI controller, which adapts the timer overflow period in the same way as for the primary side. The interrupt timestamp measurements are also made using capture/compare channels.

#### 2.3.3. Phase Offset and Cable Delays

Internal delays between the connectors and the integrated circuits, such as transmission lines on the printed circuit board, level-shifters, filters and the low-noise amplifier, have been measured and are compensated in the firmware.

However, external delays, for example, due to cables carrying PPS signals, can vary between setups. To remove setup-dependent external delays, the system was calibrated using an RF transmission over a coaxial cable of 75 m electrical length with one node placed in an RF shielding box to avoid coupling over the air between the two nodes. The PPS Out of the two devices can be measured with an oscilloscope at the cable ends to determine the time difference. This difference can be configured and compensated in one of the two devices.

#### 2.3.4. Communication Protocol

The devices of the wireless time synchronization system use a simple protocol to identify and to help to set up the required wireless links. They randomly transmit beacon messages every 3 to 5 s containing a unique device address. These beacon messages combined with a received signal power measurement within the LoRa transceiver can be used to check the antenna setup and wireless link quality. The communication and ranging/synchronization exchanges are performed in the 2.4 GHz ISM frequency band at a bandwidth BW=812 kHz and the LoRa spreading factor SF10, which is a compromise between time-of-flight resolution and range based on the specified sensitivity of the SX1280 [20]. Using BW=812 kHz obtains only half the distance resolution compared to BW=1625 kHz, but has a 6 dB higher sensitivity, which leads to a higher SNR and improved accuracy for timestamp measurements.

The primary device first sends a start packet as shown in Figure 3, which contains the target address, and it then switches to the ranging mode to perform the two sequential two-way ranging exchanges. It finally switches back to the data transmission mode and sends the timestamp values $t_{2,1}$ and $t_{3,1}$ together with the normalized clock frequency $\delta_{1}$ to the secondary side if the exchange could successfully be finalized. Additionally, not shown in Figure 3, a UNIX timestamp in seconds is sent to the secondary side. This procedure is performed every second, 700 ms after the timer overflow event, and takes about 200 ms.

## 3. Results

### 3.1. Setup

**Figure 5:**
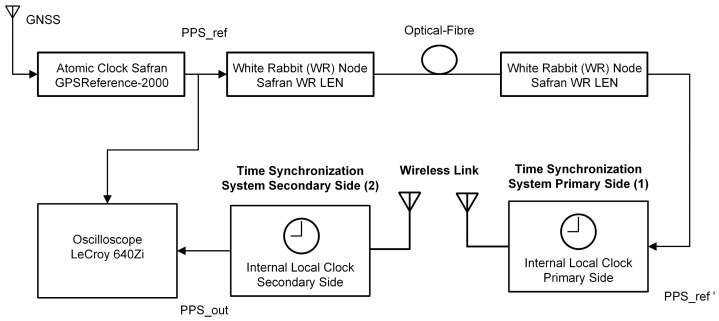
Field test setup.

The performance of the time synchronization system was assessed with a GPS-disciplined atomic clock and a WR link for transfer of the reference time as illustrated in Figure 5. The reference time in the form of a 1 Hz PPS signal (PPS_ref) is transferred to the primary synchronization device over a WR optical fiber link having a maximal time deviation (TDEV) of 30 ps over the measurement interval. Compared to a setup with two separate atomic clocks (one at either side of the time synchronization system), this setup does not suffer from phase drifts of the atomic clocks due to temperature changes. The reference time signal PPS_ref’ is then transferred by the time synchronization system over the LoRa RF link back to the secondary synchronization device, where the synchronized 1 Hz PPS time signal PPS_out is compared to the atomic clock time pulse on an oscilloscope. The time differences are then recorded on the oscilloscope to calculate the TDEV.

**Figure 6:**
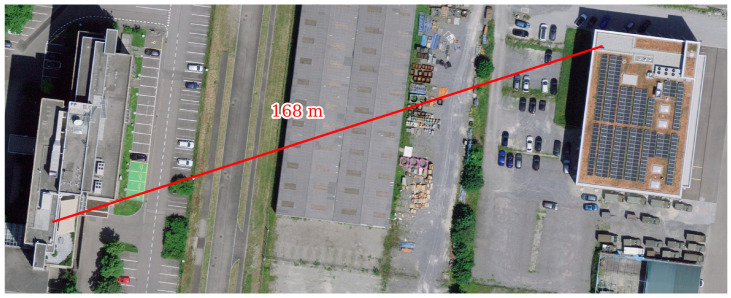
Field test with wireless link setup between two buildings (source: Federal Office of Topography swisstopo).

The time synchronization system is set up with a line-of-sight link distance of about 170 m between the roofs of the two buildings shown in Figure 6. There was additionally several meters of coaxial RF cable between the antennas and time synchronization system devices. In the first setup, two 10 dBi directional patch antennas were used on both sides of the wireless link. In the second setup, the primary side used the same 10 dBi directional patch antenna while the secondary side used an omni-directional rod antenna with a gain of 2 dBi. Even though no channel measurements were conducted, in the second setup, a more pronounced multipath propagation is expected compared to the first setup. The equipment was set up in a weather-protected environment at both ends (container room at the secondary side and a metal box at the primary side), but without temperature control.

### 3.2. Results Time Synchronization Performance

**Figure 7:**
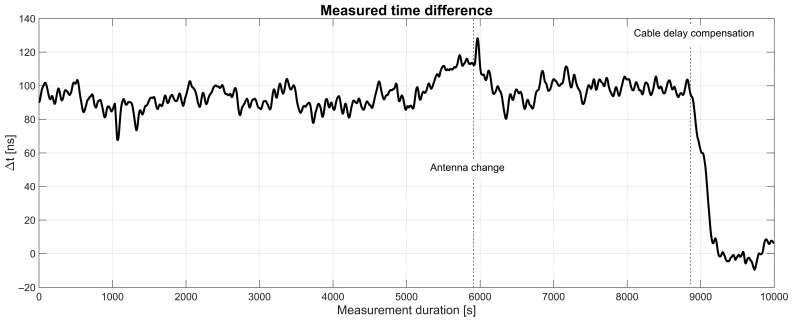
Measured time differences.

Figure 7 shows the measured time differences between the 1 Hz PPS signals PPS_ref and PPS_out after a few minutes of operation to allow for the stabilization of the control algorithm. The measurement was started without the compensation of external cable delays, leading to a considerable time difference of about 90 ns until nearly 9000 s. At this point, the external PPS cable delays were manually configured, which compensates the time difference.

The first 5900 s was recorded with the first setup using patch antennas at both sides of the wireless link, at which point the antenna on the secondary side was swapped for an omni-directional rod antenna. This second setup was active starting at 6000 s. It can be observed from Figure 7 that there is no obvious change in the behavior of the time difference when using the second setup with an omni-directional antenna on one side.

**Figure 8:**
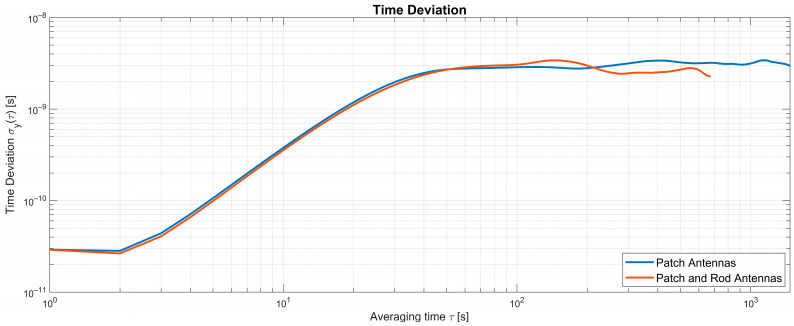
Measured time deviation (TDEV).

Figure 8 shows the TDEV for setup 1 (patch antennas) and setup 2 (patch and omni-directional rod antenna), which was calculated using Stable32 [27] according to the National Instggitute of Technology’s Handbook of Frequency Stability Analysis [28]. The deviations for 1 s intervals are 30 ps and increase to 3 ns in the average time interval from 2 s to 60 s. The deviations for both setups reach a plateau at around 3 ns for longer average times. Both setups obtain comparable performance, even though a stronger multipath propagation is expected with setup 2 compared to setup 1. This suggests that the system has a certain robustness against multipath propagation, which could be explained by the fact that the time synchronization system only needs to compensate the time of flight as measured by the system, while it is not required to resolve the individual propagation paths. The small TDEV difference between the two setups may partially be also due to the lack of a temperature-controlled environment for the equipment.

A separate measurement of one-day duration showed that the TDEV remains constant at a plateau of around 3 ns.

### 3.3. Operation over Large Distances

An early version of the time synchronization system has been operated with reliable ranging exchanges over a wireless link distance of 14 km with a line of sight between the primary and secondary side and using directional antennas with a gain of 24 dBi. By reducing the transmit power, it is possible to operate the link within the 10 dBm effective isotropic radiated power (EIRP) limit for devices without spectrum-sharing mechanisms operating in the 2.4 GHz ISM frequency band in Europe [23]. However, since the Analog Devices AD9545 jitter cleaner had not been implemented yet and no WR link for accurate transfer of the reference time was available, comparable time synchronization performance measurements were not possible. Nevertheless, this test indicates that time synchronization over distances of several kilometers is feasible.

### 3.4. Conclusions

The measured time deviations between the two synchronization devices were below 3 ns for a 170 m free-space link operating for one day. The time deviation for short intervals reduced to 30 ps for one-second intervals, which is mainly due to the frequency stability of the OCXO combined with the Analog Devices AD9545 jitter cleaner. The combination of a LoRa-based time synchronization and an adequate PLL loop circuit enabled the distribution of time information in the ns range. This is a significantly higher accuracy compared to previous work on time synchronization with LoRa modulation, which has reported accuracies in the µs range.

The channel occupancy amounts to about 20% and allows for more frequent synchronization exchanges, which would be beneficial for applications with mobile devices, and enables a multi-node network with up to five secondary devices in a star topology network without incurring accuracy losses due to time synchronization over multiple hops.

## 4. Discussion

This work demonstrates the usage of the low-power LoRa communication protocol to synchronize two devices with ns accuracy. The experimental wireless time synchronization system could be used in sensor networks for the synchronization of nodes and additionally for the transfer of small data volumes. The wireless approach is particularly suited to mobile applications or in terrain where cables are difficult to lay. The measured time deviation of 3 ns equals a range error of less than 1 m of wireless propagation, which makes it suitable for radio localization.

Table 1 provides a benchmark comparison between different time synchronization methods. GNSS-based synchronization obtains a TDEV of 1.5 ns (τ=100 s), using post-processing to remove the effect of the limited time quantization of the PPS pulse provided by the u-blox ZED-F9T module used in [3]. The optical-cable-network-based WR technology achieves the lowest TDEV, with 3 ps in the setup in [29] for τ=100 s. For the UWB system in [9], a standard deviation of 840 ps is reported, but no TDEV values. Our proposed system obtains an accuracy of 3 ns for τ=100 s, which is twice the value for GNSS-based synchronization. Compared to the simple deployment of GNSS synchronization, our system also has a higher deployment complexity and energy consumption, but the advantage is that it can be operated independently from other systems, as is the case with WR and UWB synchronization. However, UWB systems have a range of only a few tens of meters, while WR requires an optical cable network.

The deployment complexity for GNSS time synchronization is low since only the installation of an outdoor GNSS antenna is required. For WR, the deployment requires a Synchronous Ethernet (SyncE) network and an external reference clock for the primary side, while, for UWB, the deployment requires an external reference clock for the primary side. Finally, for our proposed system, an outdoor antenna and an external reference clock at the primary side is required.

Considering the obtained TDEV of 3 ns for τ=100 s for our system, we assume that a large contributor is the ranging engine in the SX1280 transceiver, which has a reported best-case standard deviation equivalent to 1.3 ns, as described in Section 1.

The implementation of encryption and authentication protocols, which are well established in LoRaWAN links [30], may be used for secured operation. This is in contrast with GNSS-based systems, which often rely on the unencrypted GPS and Galileo L1 signals.

The presented system uses power-intensive PLL chips and oven-controlled oscillators to create a frequency-stable reference clock. However, this is not suitable for low-power devices. To reduce the power consumption, unheated oscillators and a simpler clock filter are needed, which could lead to higher time deviations. This may partially be compensated with improved oscillator drift and noise modeling. Oscillator modeling can also be used to minimize the number of synchronization exchanges and adapt to predicted timestamp uncertainty.

Considering other applications, mesh networks could benefit from accurate time to optimize the channel access and active/sleep timing slots. Additionally, the security of wireless networks could be improved by tightening timing restrictions against replay attacks.

The presented time synchronization system was optimized for static line-of-sight links without time-varying multipath fading. The extension of the system to mobile time synchronization would have practical applications, such as the time synchronization of equipment in vehicles. However, in such applications, the channel would be time varying, and the coherence time may be below the duration of one time synchronization exchange. Future work could therefore optimize the physical layer modulation and protocol for applications in mobile time synchronization.

## Figures and Tables

**Table 1:** Benchmark comparison of different time synchronization methods based on TDEV with average durations τ = 1 s and 100 s.

Technology	TDEV	Power Consumption (One Node)	Deployment Complexity
GNSS [3]	200 ps (τ=1 s) 1.5 ns (τ=100 s)	0.26 W	low
WR ^1^ [29]	30 ps (τ=1 s) 3 ps (τ=100 s)	7.5 W	high
UWB [9]	No TDEV reported (standard deviation σ=840 ps)	0.7 W	medium
Proposed LoRa system	30 ps (τ=1 s) 3 ns (τ=100 s)	3.0 W	medium-high

^1^ A 50 km fiber connection between two WR nodes.

## Data Availability

The original contributions presented in this study are included in the article material. Further inquiries can be directed to the corresponding author.
